# Polarity of CD4+ T cells towards the antigen presenting cell is regulated by the Lck adapter TSAd

**DOI:** 10.1038/s41598-018-31510-6

**Published:** 2018-09-06

**Authors:** Greger Abrahamsen, Vibeke Sundvold-Gjerstad, Meseret Habtamu, Bjarne Bogen, Anne Spurkland

**Affiliations:** 1 Department of Molecular Medicine, Institute of Basic Medical Sciences, University of Oslo, Oslo, Norway; 2 Armauer Hansen Research Institute, Addis Abeba, Ethiopia; 3 Centre for Immune Regulation, Institute of Immunology, Oslo University Hospital, Rikshospitalet, Oslo, Norway; 4 K. G. Jebsen Centre for Influenza Research, University of Oslo, Oslo, Norway

## Abstract

Polarization of T cells towards the antigen presenting cell (APC) is critically important for appropriate activation and differentiation of the naïve T cell. Here we used imaging flow cytometry (IFC) and show that the activation induced Lck and Itk adapter T cell specific adapter protein (TSAd), encoded by *SH2D2A*, modulates polarization of T cells towards the APC. Upon exposure to APC presenting the cognate antigen Id, *Sh2d2a*−/− CD4+ T cells expressing Id-specific transgenic T cell receptor (TCR), displayed impaired polarization of F-actin and TCR to the immunological synapse (IS). *Sh2d2a*−/− T-cells that did polarize F-actin and TCR still displayed impaired polarization of PKCξ, PAR3 and the microtubule-organizing center (MTOC). *In vitro* differentiation of activated *Sh2d2a*−/− T cells was skewed towards an effector memory (Tem) rather than a central memory (Tcm) phenotype. A similar trend was observed for Id-specific TCR *Sh2d2a*−/− T cells stimulated with APC and cognate antigen. Taken together our data suggest that TSAd modulates differentiation of experienced T cells possibly through polarization of CD4+ T cells towards the APC.

## Introduction

Upon stimulation of T cells via TCR and other surface receptors, initiation of signalling cascades eventually results in proliferation and differentiation into various T cell phenotypes^1^. The molecular details for how signalling in T cells is controlled after the initial triggering of the TCR is not fully understood.

TCR binding to its cognate antigen-MHC complex on antigen presenting cells may result in formation of an immunological synapse (IS) at the interface between the T cell and the APC. IS formation is dependent on localized intracellular signals that results in cytoskeletal and membrane reorganization towards the contact site (reviewed in^2^). Polarisation of actin, TCR and signalling molecules towards the IS are required for proper activation and function of T cells, where CD4+ T cells engage with the APC for several hours^1^. Sustained TCR signalling is subsequently maintained through recycling TCR and Lck to the IS during activation^3,4^, until signal termination by negative feedback mechanisms or TCR degradation^5,6^. Providing a critical regulatory stimulus, the IS facilitates differentiation of T cells into Tem or Tcm subsets,^1,7,8^.

Interruption of IS formation may skew or disrupt CD8+ T cell differentiation^9–12^. Upon triggering of the TCR, formation of the IS initially involves reorganising the actin cytoskeleton towards the cell interface, followed by movement of TCR micro-clusters towards the centre of the IS (cSMAC). As the IS matures, polymerized actin reorganises and relocates to the periphery (dSMAC) while microtubule organizing centre (MTOC) reorients to a position beneath the IS (reviewed in^13^).

A novel player in regulation of T cell polarity, may be the cytosolic Lck and Itk adapter TSAd^14^, encoded by *SH2D2A*. We recently found that lack of TSAd is associated with reduced accumulation of F-actin at the interphase between CD4+ T cells and APC^14^. TSAd is induced in both CD4+ and CD8+ T cells upon engagement of the TCR^15,16^. However, the role of TSAd in activated T cells is only partially understood. TSAd affects proximal T cell signalling events via its interaction with Lck^16,17^, possibly by promoting phosphorylation of Lck Tyr^192^ by Itk^14^. TSAd also regulates chemokine induced T cell migration and actin polymerization via its interaction with Itk^18^.

Despite TSAd being involved in TCR signalling, unchallenged *Sh2d2a*−/− mice display only minor alteration in overall immune phenotype^15,19,20^. TSAd may influence specific NK cell immune responses, since *Sh2d2a*−/− mice displayed reduced clearance of a mutant murine cytomegalovirus which does not activate the NK cell receptor Ly49H^21^. Recently, we found that TSAd affects CD4+ T cell-mediated rejection of experimental multiple myeloma^19^. Wild type mice are highly susceptible to MOPC315 myeloma, while mice carrying transgenic TCR, recognizing a peptide (Id) derived from the variable region of the L chain of the MOPC315 IgA (Id-specific TCR), are partially protected^22^. The Id-peptide is presented on I-E^d^ by tumor-infiltrating macrophages. Rejection is caused by interplay of Th1 T cells and M1-activated macrophages^23^. The *Sh2d2a*−/− Id-specific TCR mice displayed increased resistance towards myeloma^19^, however the molecular mechanism for the increased resistance remains to be determined.

We here examined the hypothesis that TSAd regulates T cell differentiation through altered polarization of T cells towards APC. Using imaging flow cytometry (IFC), the distribution of signalling molecules towards the IS in T-cell/APC conjugates was assessed^19^. We found that in Id-specific TCR T cells, TSAd affected actin polymerization as well as polarization of TCR, PKCξ and MTOC at the IS. Our results suggest that altered polarization of T cells may influence differentiation of effector and memory T cells.

## Results

### TSAd is continuously expressed in activated T cells

**Figure 1:**
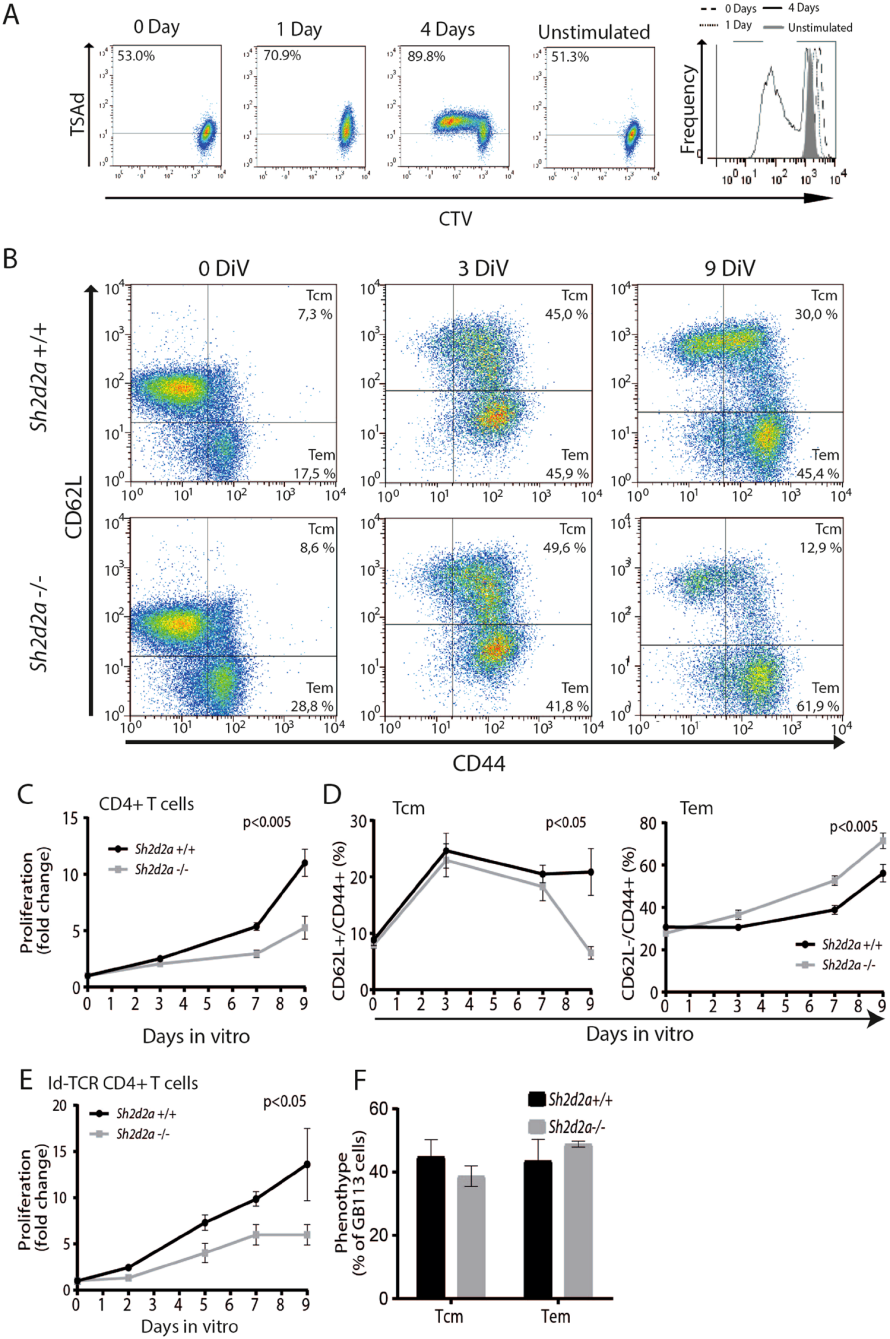
TSAd impact on proliferation and phenotype of CD4+ T cells. (**A**) TSAd is expressed in proliferating human CD4+ T cells upon anti-CD3/CD28 stimulation. Dot plots show TSAd expression in relation to CTV dilution, while histogram shows degree of CTV dilution over 4 days with or without stimulation. (**B**) Dot plots show expression of CD62L and CD44 in *Sh2d2a*+/+ and *Sh2d2a*−/− mouse CD4+ T cells from C57/BL6 mice upon anti-CD3/CD28 and IL2 stimulation, over 9 days *in vitro*. (**C**) Accumulated proliferation of CD4+ T cells from *Sh2d2a*+/+ and *Sh2d2a*−/− mice stimulated as in (B). (**D**) Relative proportion of Tcm and Tem cells in T-cell cultures stimulated as in (**B**). Data in C and D represent mean +/−SD of three independent experiments. Statistical significance was assessed by two-way ANOVA. E) Accumulated proliferation of Id-specific TCR transgenic CD4+ T cells from *Sh2d2a*+/+ and *Sh2d2a*−/− mice upon culture under Th1 skewing conditions (peptide loaded APC in presence of IL12 and anti-IL4). Data represent mean +/− SD of three independent experiments. Statistical significance was assessed as in C and D. (**F**) Graph shows the frequency of GB113+ T cells with Tcm or Tem phenotype. Data in G and H represents mean +/− SD of 3 independent experiments, analysed using paired student’s t-test.

We previously demonstrated increased resistance towards experimental myeloma in the absence of TSAd^19^. Resistance towards myeloma in this model is driven by tumor-specific Th1 cells which activate tumor-infiltrating antigen-presenting macrophages^24,25^. TSAd expression is induced in T cells upon activation through the TCR/CD3 complex^15,16^. To begin to explore the molecular mechanism for increased resistance to myeloma in the absence of TSAd, we thus first asked whether TSAd continues to be expressed in proliferating T cells. The amount of TSAd in CD3/CD28 activated human CD4+ T cells was measured in dividing cells using CTV dilution combined with intracellular staining for TSAd. TSAd was found to be expressed at the same level over several generations of proliferating human CD4+ cells (Fig. 1A) indicating that this intracellular Lck-adapter potentially could affect T cell proliferation, differentiation or cell survival.

### TSAd promotes differentiation of Th1 cells with a central memory phenotype

Having found that TSAd is continuously expressed in proliferating T cells, we next asked whether TSAd affects the proliferation and phenotype of activated CD4+ T cells. Previous studies of short term stimulated TSAd deficient T cells have revealed conflicting results^15,18,20^. Here we stimulated murine *Sh2d2a*+/+ or *Sh2d2a*−/− CD4+ T cells from C57/BL6 mice for three days using anti-CD3/anti-CD28 activator beads as a surrogate for APC, followed by incubation with IL-2 for six days to promote cytokine mediated proliferation^26^ (Fig. 1B and C). Live cells were counted at the indicated times, and the frequency of live cells expressing CD44 and CD62L was analysed by flow cytometry in order to assess the size of the T cell populations with a central memory phenotype (i.e. Tcm or CD44high/CD62Lhigh) and an effector memory phenotype (i.e. Tem or CD44high/CD62Low) (Fig. 1B and C). Over the nine days of IL-2 stimulation, the accumulated proliferation of *Sh2d2A−/−*CD4+ T cells was significantly lower than that of *Sh2d2a*+/+ CD4+ T cells (Fig. 1C). Moreover, the cultured *Sh2d2a*−/− CD4+ T cells were significant skewed towards a Tem phenotype as compared to *Sh2d2a*+/+ CD4+ T cells (Fig. 1D). Similarly, *Sh2d2a*−/− Id-specific TCR transgenic CD4+ T cells^19,27^ cultured under Th1 stimulating conditions in the presence of Id-peptide loaded APCs from *Sh2d2a*+/+ spleens^22^, displayed a significantly reduced accumulated proliferative response compared to the corresponding *Sh2d2a*+/+ Id-specific TCR CD4+ T cells (Fig. 1E). These same cells showed a trend towards skewed Tem differentiation (Fig. 1F). Taken together, these data suggests that TSAd influences cytokine mediated proliferation and differentiation of T cells.

### TSAd regulates polarization of F-actin in antigen stimulated CD4+ T cells

**Figure 2:**
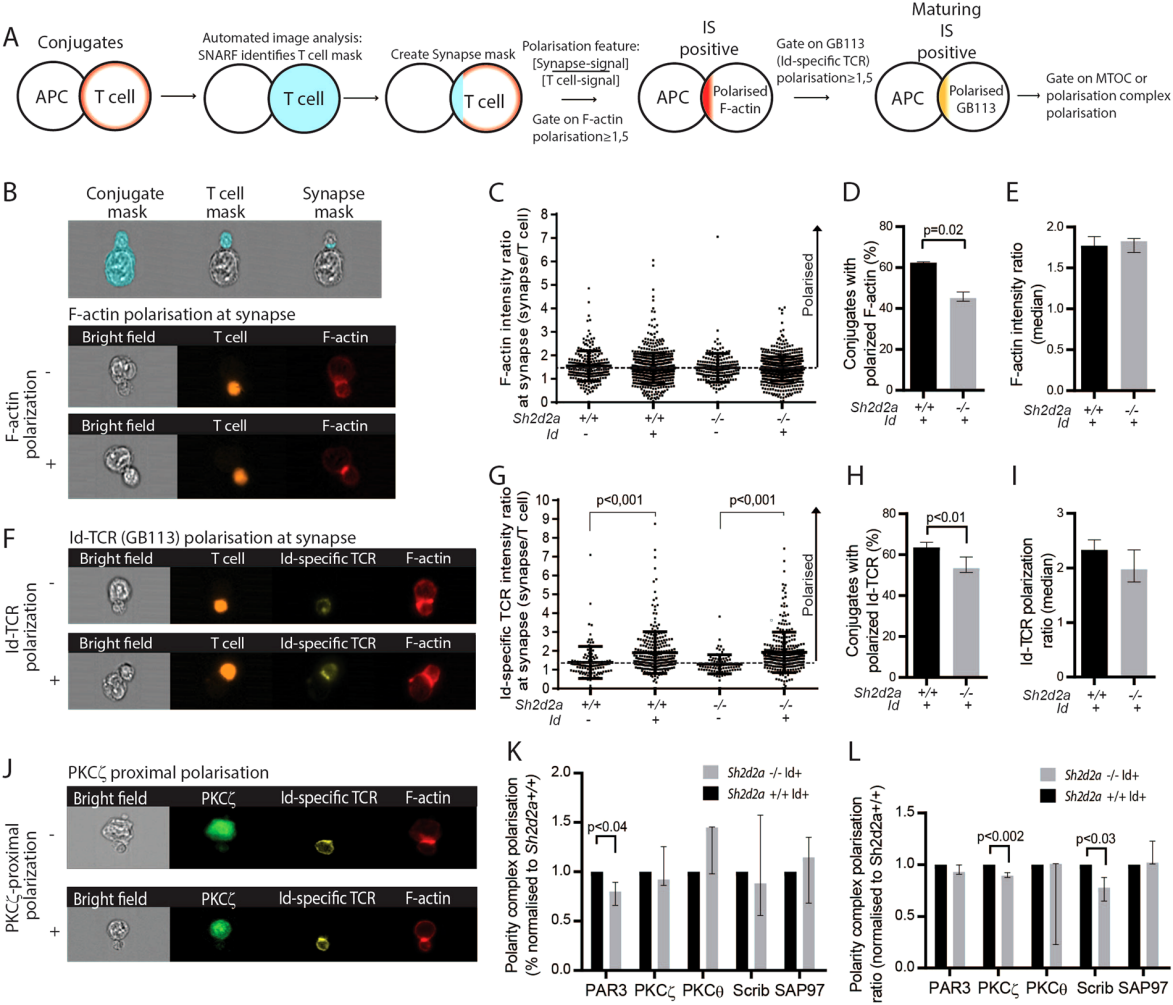
TSAd affects polarization of actin, TCR and polarity complex proteins at the IS. Murine Id-specific TCR-transgenic CD4+ T cells were incubated for 30 minutes in the presence of Id negative (A20 cells) or Id positive (F9 cells) APC, prior to staining with the indicated markers and analysis of T cell:APC conjugates by IFC. (**A**) Conjugates are identified as a single SNARF^+^ T cell in association with an unlabeled APC. A T cell mask is applied together with a synapse mask. The polarization feature is determined as the signal intensity in the synapse area relative to that of the entire T cell. A value >1.5 implies that the marker is polarized towards the APC. Conjugates where F-actin in T cells is polarized towards the APC is defined as IS positive. IS-positive conjugates with polarized GB113^+^ (Id-specific TCR) are defined as maturing IS-positive. Conjugates with a maturing IS are then analyzed for polarization of other markers, as indicated in the text and figures.) Masking strategy was employed to identify the T cell synapse in T cell:APC conjugates. Sample images show absence (−) and presence (+) of F-actin polarization towards IS in Id-specific T:APC conjugates. (**C**) Histogram shows ratio of F-actin intensity values at synapse of individual *Sh2d2a*+/+ or *Sh2d2a*−/− Id-specific TCR CD4+ T cells interacting with Id negative A20 cells or Id positive F9 cells (all analyzed conjugates from one representative experiment). The polarization ratio was calculated by dividing the F-actin signal in the synapse mask with the F-actin signal in the entire cell. The stippled line indicates the threshold value of 1.5. Conjugates with polarization ratio >1.5 were defined as having F-actin polarized to the IS. (**D**) Graph shows frequency of Id-specific T:APC conjugates displaying F-actin polarization ratio >1.5. (**E**) Graph shows median F-actin intensity ratio values in Id-specific T:APC conjugates in the presence of Id positive F9. Data in C and D represents median values (+95% CI) of three independent experiments, paired student’s t-test. (**F**) Polarization of Id-specific TCR (staining with TCR clonotype-specific GB113 mAb) in conjugates displaying F-actin polarization. Image gallery shows conjugate without (−) and with (+) Id-specific TCR polarization. (**G**) Histogram shows Id-specific TCR intensity values (calculated as for F-actin above) at synapse of individual *Sh2d2a*+/+ or *Sh2d2a*−/− Id-specific TCR CD4+ T cells in conjugate with Id negative or positive APC as in (**B**) and gated for polarization of F-actin (all analyzed conjugates from one representative experiment). Stippled line defines threshold for identification of conjugates with Id-specific TCR polarized to IS. Non-parametric Mann-Whitney test. (**H**) Graph shows frequency of Id-specific T:APC conjugates with Id-specific TCR polarized to the synapse. (**I**) Graph shows median Id-specific TCR polarization ratios towards synapse in Id-specific T:APC conjugates. Data in G and H represent median values (+95% CI) of three independent experiments, analyzed using paired student’s t-test. (**J**) Image gallery showing polarization of PKCξ towards synapse upon engagement of TCR. (**K**) Graph shows relative frequencies of Id-specific T: -APC conjugates with polarization of indicated polarity complex molecules towards IS relative to *Sh2d2a*+/+. (**L**) Graph show relative values of the intensity ratios in Id-specific T:-APC conjugates of the indicated polarity complex molecules. (**j**) and (**K**) Data represents the median values (+95% CI) normalized to those observed in *Sh2d2a*+/+ Id-specific T: APC conjugates of three independent experiments, n >50 gated events in each experiment. Statistical significance was assessed using paired student’s t-test.

Asymmetric distribution of signalling molecules in APC-conjugated T cells have previously been associated with subsequent differentiation of T cells into Tcm and Tem cells^8^. TSAd regulates actin polymerization at the interphase between T cell and APC^14^. As TSAd is continuously expressed in activated T cells over multiple cell divisions, and since cytokine mediated proliferation of *Sh2d2a*−/− T cells is diminished compared to *Sh2d2a*+/+ T cells, we thus asked whether TSAd affects the polarization of critical molecules towards the IS in experienced T cells. Briefly, to generate IS-positive Id-specific T:APC-conjugates *in vitro* Id-specific TCR CD4+ T cell blasts were used to ensure adequate expression of TSAd (Fig. 1A) and its interaction partner Itk^14^. SNARF-labeled T cells were co-incubated with A20 lymphoma cells or F9 lymphoma cells expressing Id-peptides bound to I-E^d^ (F9 is a derivative of A20) as APC for 30 minutes (for details see Materials and Methods). After staining with Id-specific TCR specific antibody and phalloidin labelling F-actin, cells were analysed by IFC. T-cells conjugated to APC were examined for polarisation of F-actin to the IS (Fig. 2A and B). Polarisation of F-actin was determined by comparing the signal in the synapse mask to the signal in the whole cell (see Materials and methods for details). In line with our previous finding^14^, significantly fewer conjugated *Sh2d2a*−/− T cells polarised F-actin to the IS compared to *Sh2d2a*+/+ T cells (Fig. 2C and D). However, among conjugated T cells displaying polarized F-actin, the degree of polarized F-actin, as assessed by the median polarisation ratio, was no significant difference in *Sh2d2a*−/− compared to *Sh2d2a*+/+ T cells (Fig. 2E).

### TSAd regulates polarization of TCR to the IS

Presence of polarized F-actin on the T cell side of the Id-specific T:APC contact area was defined as a marker for IS-positive T cells. TCR is known to accumulate at the centre of the mature IS^13^. We thus considered that accumulation of the Id-specific TCR at the IS may be a marker for a maturing synapse. When analysing IS-positive Id-specific T:APC conjugates, formed after 30 minutes of co-incubation of T cells with APC, for accumulation of TCR at the IS (Fig. 2F), we found that IS-positive *Sh2d2a*−/− T cells displayed significantly fewer Id-specific T:APC conjugates with polarized TCR than their *Sh2d2a*+/+ counterpart (Fig. 2G and H). However, although the median polarisation ratio of TCR also was lower in *Sh2d2a*−/− T cells compared to *Sh2d2a*+/+ T cells displaying a maturing IS, this difference did not reach statistical significance (Fig. 2I). No significant differences in the frequency of T-APC conjugates with polarized F-actin or TCR was observed between *Sh2d2a*+/+ and *Sh2d2a*−/− T cells when A20 cells lacking Id was used as APC (Fig. 2C and G and data not shown). Taken together, these results indicate a deficiency of IS maturation in the absence of TSAd.

### TSAd regulates polarization of polarity proteins in T cells

T cell polarity^28–32^ is controlled by highly conserved intracellular polarity proteins known as the Scribble (including Scrib and SAP97) and PAR3 (including PAR3, PKCξ and PKCθ) protein complexes respectively. These polarity complexes define distinct spatial regions of the cell through differential localisation of macromolecules. There is accumulating evidence that the PAR3 complex modulates T cell polarisation, migration and IS formation^29–31,33,34^. In order to examine whether polarisation complexes were affected by the absence of TSAd, we analysed Id-specific T:APC conjugates with a maturing IS (displaying accumulation of F-actin and TCR) for distribution of PAR3, PKCξ, PKCθ, Scrib or SAP97 (Fig. 2J). In Id-specific T:APC conjugates having a maturing IS after 30 minutes of co-incubation of T cells with APC, there was no significant difference in the frequency of conjugates displaying asymmetric distribution of polarity complexes within the T cell (Fig. 2K). However, the proportions of PKCξ and SCRIB polarised towards the synapse in the conjugates, as measured by their median polarisation ratios, were significantly lower in *Sh2d2a*−/− than in *Sh2d2a*+/+ T cells (Fig. 2L). In Id-specific T:APC conjugates involving A20 cells, there were no significant differences (data not shown).

### TSAd promotes re-localization of MTOC to the maturing IS of CD4+ T cells

**Figure 3:**
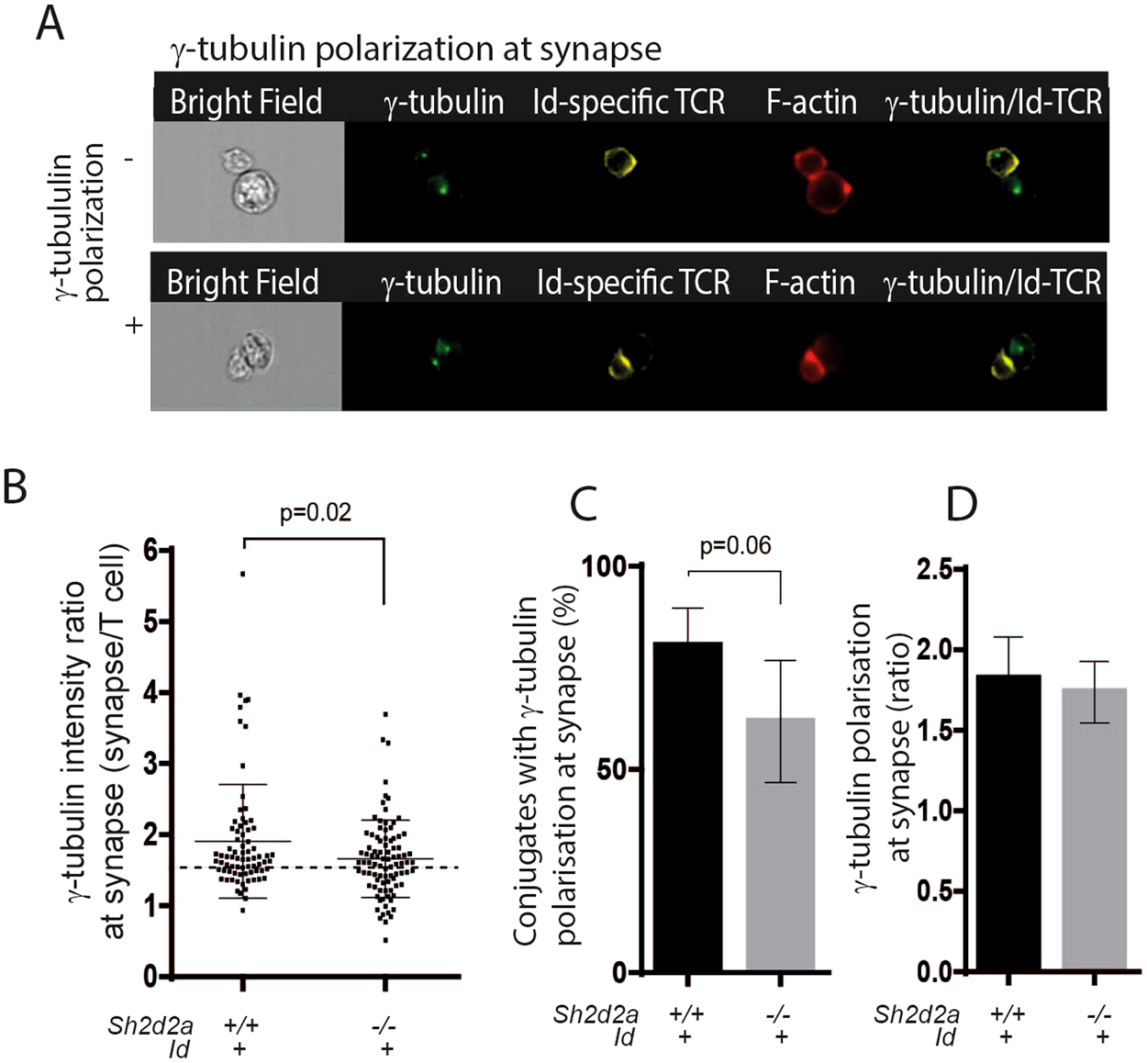
Polarization of γ-tubulin towards IS in CD4+ T cells upon engagement of TCR. (**A**) Sample images show absence (−) and presence (+) of γ-tubulin oriented towards IS in Id-specific T cells conjugated to Id positive F9 cells, displaying polarized F-actin and Id-specific TCR. (**B**) Histogram shows γ-tubulin polarization ratio in Id-specific T:APC conjugates displaying F-actin and Id-specific TCR polarization to the synapse, using T cells from either *Sh2d2a*+/+ or *Sh2d2a*−/− mice, after 30 minutes incubation. The stippled line indicates the threshold value of 1.5. Conjugates with polarization ratio >1.5 were defined as having γ-tubulin polarized to IS. Data are from one experiment. Statistical significance was assessed using Mann-Whitney test. (**C**) Graph shows frequency of Id-specific T:APC conjugates displaying γ-tubulin polarization to the synapse. (**D**) Graph shows median γ-tubulin polarization ratios towards synapse in the presence of Id positive F9 cells. Data in C and D represent median values (+95% CI) of three independent experiments, n > 50 gated events in each experiment. Statistical significance was assessed using paired student’s t-test.

The relocation of MTOC to beneath the interface between the T cell and the APC is a hallmark of a mature IS^13^. Having found that *Sh2d2a*−/− T cells displayed reduced frequency of conjugates with polarised F-actin, TCR and reduced polarisation of the PAR3 complex member PKCξ to the IS, we went on to assess the relocation of the MTOC in Id-specific T:APC conjugates. T cells and APC’s were co-incubated for 30 minutes, followed by visualization of MTOC by intracellular staining for γ-tubulin. The maturing IS was defined as previously described, using phalloidin and anti-TCR staining of the conjugates (Figs 2A and 3A). The intensity ratio of γ-tubulin at the synapse was assessed as previously described (Figs 2A, 3B and Materials and methods).The frequency of conjugates displaying MTOC re-localisation beneath the IS in T cells was significantly lower in *Sh2d2a*−/− compared to *Sh2d2a*+/+ T cells (Fig. 3C). However, once the MTOC was polarised, the proportion of γ-tubulin signal relocating to the IS was not significantly different in *Sh2d2a*−/− T cells compared to that observed in *Sh2d2a*+/+ (Fig. 3D).

### TSAd influences the kinetics of F-actin, TCR, PKCξ and MTOC polarisation in CD4+ T cells

**Figure 4:**
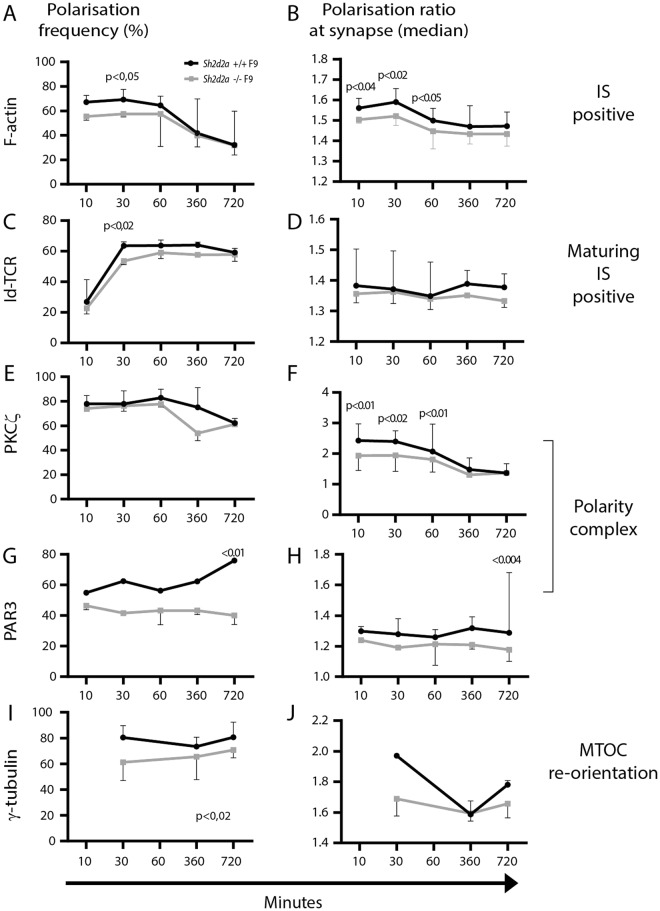
*Sh2d2a* influences the kinetics of CD4+ T cell polarization towards the APC upon TCR-engagement. (**A**,**C**,**E**,**G** and **I**) Line charts show the frequency of (**B**) polarized F-actin (IS-positive conjugates), (**C**) Id-specific TCR (maturing IS-positive conjugates), (**D**) PKCξ, G) PAR3 and I) γ-tubulin (within maturing IS-positive conjugates) in Id-specific TCR T:APC conjugates displaying polarization in T cells towards the APC. (**B**,**D**,**F**,**H** and **J**) Line charts show the corresponding median polarization ratios towards the IS of Id-specific TCR T cells displaying polarization of F-actin, Id-specific TCR, PKCξ, PAR3 or γ-tubulin respectively. Data represents the median +− range of three separate experiments, n > 50 gated events in each experiment. Significance was determined by two-way ANOVA and Sidak’s multiple comparison test.

**Figure 5:**
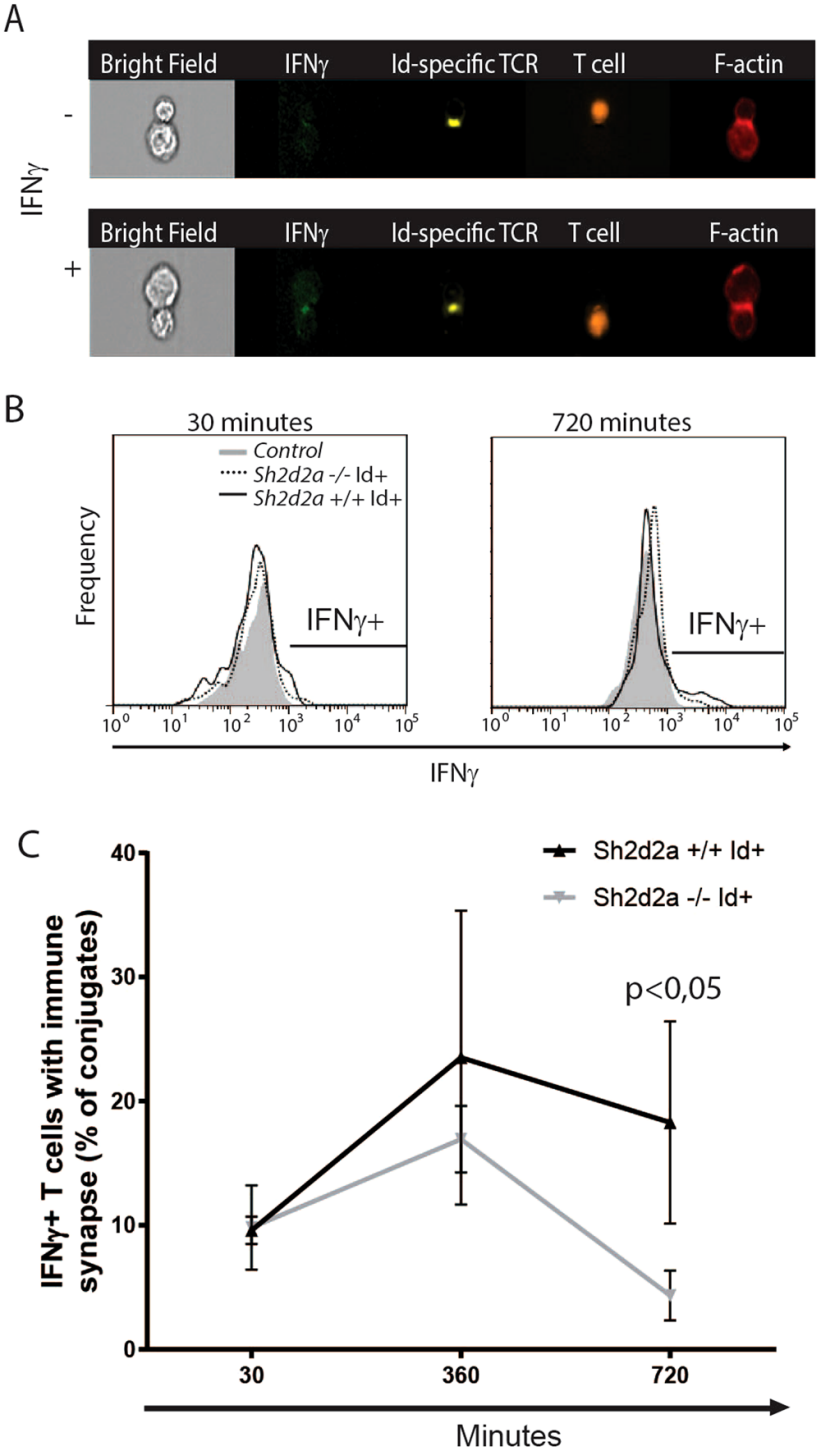
*Sh2d2a* affects IFNγ in Id-specific TCR CD4+ T cells. (**A**) Sample images show absence (−) and presence (+) of IFNγ in TCR-Id CD4+ T cells conjugated to Id-positive APC, and displaying polarized F-actin and Id-specific TCR. Images showing expression of IFNγ accumulated to the T cell synapse. (**B**) Histograms show IFNγ staining of T cells with F-actin and TCR polarisation towards the synapse when conjugated to APC for 30 and 720 minutes respectively. (**C**) Line charts showing kinetics of IFNγ polarisation to synapse in T cell conjugates upon Id presentation. Data represents the average of 3 separate experiments, n > 50 gated events in each experiment. Significance was determined by two-way ANOVA and Sidak’s multiple comparison test. Mean +/− SD.

Having found that maturation of the IS was affected in *Sh2d2a*−/− T cells early after conjugation with APC, we proceeded to analyse the kinetics of IS maturation in *Sh2d2a*+/+ and *Sh2d2a*−/− T cells over a 12 hour time course, including also the polarisation of the polarity proteins PAR3 and PKCξ (Fig. 4). As previously, T cells and Id-positive APC (F9 cells) were co-incubated for the indicated time points, followed by staining with the indicated antibodies. The initial reduced frequency of *Sh2d2a*−/− T cells displaying polarized F-actin as well as the relative amount of F-actin polarized to the IS in conjugated *Sh2d2a*−/− T cells was not observed at later time points (Fig. 4A and B). Similarly, the difference in frequency of TCR polarised conjugates towards IS was not observed at later time points (Fig. 4C). As already noted, the relative amount of TCR accumulated towards the synapse was not significantly different at early time points and remained so throughout the entire time course (Fig. 4D). The frequency of conjugates with PKCξ polarised towards the mature IS was similar in *Sh2d2a*−/− and *Sh2d2a*+/+ T cells over the course of the experiment (Fig. 4E), while the reduced amount of polarized PKCξ in *Sh2d2a*−/− was only observed for up to 60 minutes of Id-specific T:APC co-culture (Fig. 4F). Both the frequency of conjugates with PAR3 at the IS, as well as the amount of PAR3 polarised towards the mature IS, was significantly lower in *Sh2d2a*−/− only at the latest time point tested (720 minutes) (Fig. 5G and H). The frequency of conjugates with polarized γ-tubulin was significantly lower in *Sh2d2a*−/− over the course of the experiment (Fig. 4I). However once polarisation occurred, γ-tubulin was similarly polarised as assessed by polarisation ratio, irrespective of TSAd (Fig. 4J). There were no significant differences in polarisation in the absence of Id presentation (data not shown). Taken together, these results support a role for TSAd in regulating maturation and organisation of the IS at early time points during initial T cell:APC interaction.

### IFNγ is reduced in antigen stimulated Sh2d2a*−/−*Id-specific TCR T cells

Cytokine secretion is a crucial part of T cell effector function. Several cytokines are polarised and secreted through the IS, including IFNγ^35^. Polarisation of IFNγ to the IS is dependent on polarisation of F-actin, but not MTOC reorientation^35,36^. Having found that TSAd influences accumulation of polarized F-actin as well as members of the PAR3 polarity complex to the IS, we examined whether *Sh2d2a*−/− also affected IFNγ during T cell:APC interaction.

As above Id-specific TCR T blasts and APC were co-incubated for the indicated time points and subsequently stained intracellularly for IFNγ- F-actin and TCR. IFNγ expression in Id-specific TCR T cells with maturing IS was assessed (Fig. 5A). After 30 minutes incubation, both *Sh2d2a*+/+ and *Sh2d2a*−/− Id-specific T:APC conjugates displayed intracellular IFNγ (Fig. 5B,C). The number of IFNγ expressing *Sh2d2a*+/+ T cells varied throughout the experiment, while after 720 minutes of incubation significantly fewer *Sh2d2a*−/− T cells were expressing IFNγ (Fig. 5C). Taken together, the effect of TSAd on IS-formation and T cell differentiation includes a positive effect on amount of IFNγ-in antigen stimulated CD4+ T cells.

## Discussion

Polarisation of signalling molecules in the triggered T cell towards the APC is crucial for normal activation and function of T cells. Here we have shown that the Lck-adapter TSAd is required in the early phases in the T cell’s interaction with APC. Absence of TSAd results in altered differentiation and proliferation of activated T cells *in vitro*.

In this study, we mainly used imaging flow cytometry, which allows for the rapid and unbiased analysis of large numbers of Id-specific T:APC conjugates. Though the resolution of IFC is inferior to confocal microscopy, the method permits quantitative high throughput analysis of Id-specific T:APC conjugates, without encountering issues of photo-bleaching and observer bias in identifying Id-specific T:APC conjugates associated with confocal microscopy.

A key feature of TSAd is that it is rapidly induced upon activation of the T cells. Resting T cells display low levels, while recently activated T cells express high levels of TSAd. In T cells primed via inflammatory cAMP inducing signals, TSAd protein expression may be induced already 15 minutes after initial TCR triggering of primary resting T cells^37^. Here we show that expression of TSAd is stably maintained over several cell divisions, and that absence of TSAd results in reduced proliferation and a significant skewing towards effector T cell phenotype. This support the notion that TSAd also regulates T cells after the initiation of TCR stimulation. Although reduced proliferation of *Sh2d2a*−/− T cells in response to TCR-triggered activation was initially reported^15^, a significant difference in short term anti-CD3/anti-CD28 stimulated T cell proliferation has not consistently been observed^19^. Our present data strongly indicates that TSAd may impact the T cell phenotype upon long term or chronic immune challenges.

The main finding of this study is that TSAd regulates polarization of key molecules, including actin and members of the PAR3 complex, to the IS in T cells during early APC interactions. This results in altered MTOC polarisation, as well as IFNγ expression. Secretion of IFNγ at the IS is dependent on actin remodelling^36^. Intracellular vesicles associated to the GTPases Rab3d and Rab19 traffic IFNγ for secretion at the IS^35^. Whether these GTPases are also affected by the presence of TSAd remains to be studied.

Altered polarization of T cells towards the APC may represent a possible molecular mechanism for the improved resistance of *Sh2d2a*−/− Id-specific TCR transgenic mice towards experimental myeloma that we previously observed^19^. IFNγ is a major effector cytokine produced by CD4+ T cells, which can induce tumor-killing macrophages^24,25^. Elimination of the MHC negative plasmacytoma MOPC315 involves indirect recognition by CD4+ T cells of tumor antigen presented by infiltrating macrophages^25,38,39^, followed by differentiation of cytotoxic macrophages through direct contact with the T cells^39,40^. Our data suggest that altered cytoskeletal and polarisation dynamics in TSAd deficient CD4+ T cells during APC interactions could provide reduced secretion of IFNγ at the IS. It is thus possible that the improved tumor rejection in TSAd deficient Id-specific TCR transgenic T cells^19^ is caused by diffuse instead of polarized secretion of IFNγ during CD4+ T cell interactions with APC. In conjunction with a more slowly dividing effector T cells population, this may lead to improved and persistent recruitment of cytotoxic macrophages and increased tumor killing^24,38,40,41^. Whether recruitment of cytotoxic macrophages and bystander killing of the plasmacytoma cells is affected by lack of TSAd need to be explored^39,40^.

Our present data show that TSAd modulates early polarisation of the cytoskeleton as well as MTOC re-arrangement in T cells, however the molecular mechanism for how TSAd affects polarity of T cells remains to be determined. TSAd is an adapter for both Lck and Itk and promotes phosphorylation of Itk by Lck^18^ and phosphorylation of Lck Tyr^192^ by Itk^14^. T cells expressing Lck Phe^192^ displayed reduced ability to form conjugates with antigen presenting cells^14^. Lck is required for actin polarisation and MTOC reorientation upon TCR engagement^42^.

Cdc42^43,44^ is a key GTPase for establishing cell polarity^45,46^. Lck and Itk mediated signalling upon TCR stimulation is required for Cdc42-WASP controlled actin polarisation towards the IS^44^. TSAd also promotes recruitment of Lck and SLP-76 to Nck^47^. SLP-76 and Nck coordinates WASP recruitment to Cdc42^43^. Cdc42 controls polarity of cells through activation of multiple polarity pathways, including the PAR3 polarity complex^48^.

It is likely that the alterations in actin and polarity complex polarization in the *Sh2d2a*−/− T cells are mechanistically linked to the overall reduced MTOC polarisation observed in the same cells. Though the mechanism for MTOC reorientation in T cells remains unclear, it appears to involve motor protein activity and polarity complex interactions^49,50^. It was recently proposed that Arp2/3 mediated actin nucleation tethers MTOC to the nucleus, through the linker of nucleoskeleton and cytoskeleton (LINC) complex^51^. During TCR stimulation Arp2/3 is recruited to the plasma membrane, resulting in local depletion of Arp2/3 and release of MTOC from the nucleus, allowing for its reorientation towards the IS^51^.

**Figure 6:**
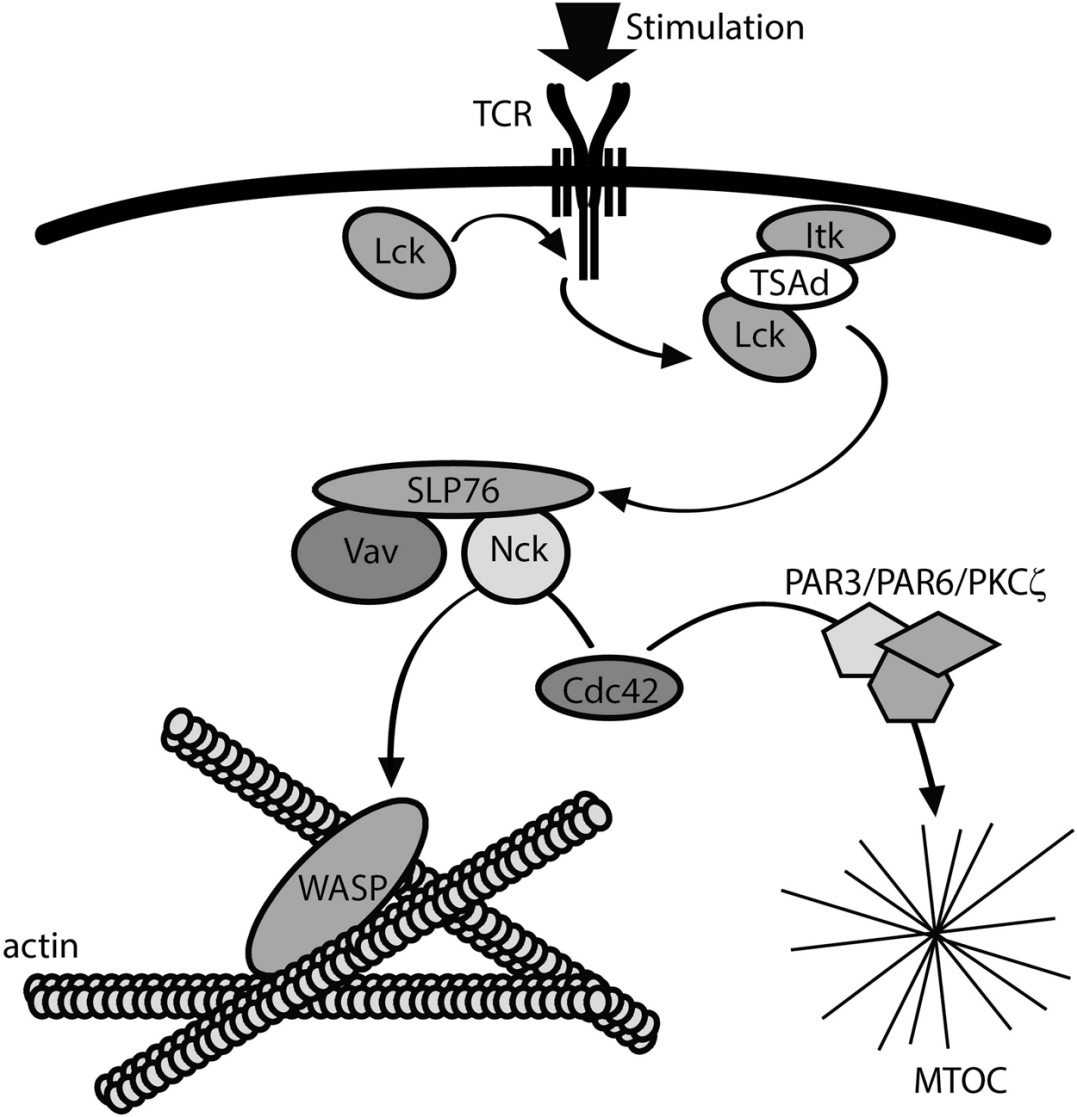
Proposed mechanism for TSAd modulation of T cell polarity upon TCR stimulation. Upon repeated TCR stimulation, TSAd is expressed and modulates the activity of Lck and Itk. Presence of TSAd affects recruitment of Lck and SLP-76 to Nck and promote downstream re-organization of the cytoskeleton and MTOC towards the IS, possibly mediated by activation of Cdc42.

Taken together, it is conceivable that the change in TCR induced polarisation dynamics observed in *Sh2d2a*−/− CD4+ T cells is ultimately linked to altered Lck signalling, and downstream Itk and Nck mediated cytoskeletal polarisation^14,47^ (depicted in Fig. 6^52,53^). However, whether TSAd modulates downstream polarization events through a Cdc42-dependent mechanism remains to be determined.

## Conclusion

Polarisation of T cells towards the APC and altered expression on IFNγ in conjugated T cells stimulated with cognate antigen is regulated by the Lck adapter TSAd. This may explain why TSAd deficiency is associated with increased resistance towards tumors where IFNγ dependent macrophage mediated cytotoxicity is crucial.

## Materials and Methods

### Ethical Statements

All experiments were performed in accordance with relevant guidelines and regulations. The animals were bred under conventional conditions, regularly screened for common pathogens and housed in compliance with guidelines set by the Experimental Animal Board under the Ministry of Agriculture of Norway. All experimental protocols involving transgenic and wild type animals were approved by the National Committee for Animal Experiments (Oslo, Norway). Peripheral blood mononuclear cells were isolated from healthy, adult, anonymous blood donors. Informed consent was obtained from all blood donors. The study was approved by the regional ethics committee (REK no S-09325b).

### Antibodies and reagents

Antibodies used were anti-human CD3ε (OKT3, American Type Culture collection, Manassas, VA), anti-CD28 (clone CD28.2, BD Biosciences), and anti-TSAd-DyLight 488 (clone OTI3C7, Origene), anti–IL-4 (11B11, Bio X Cell), anti-IFNγ-FITC, anti-CD4-PerCP-Cy5.5 (RM4–5) and anti-CD44-FITC (KM201, BD), anti-CD62L-PE (MEL-14, Southern Biotech), anti-γ–tubulin (GTU-88, Sigma) and anti- Id-specific TCR-PE (GB113^54^). Polyclonal antibodies were anti-PAR3 (Cat# 07-330, Millipore), anti-PKCξ (Η - 1), anti-PKCθ (C-18), anti-Scrib (C-6) and anti-SAP97 (H-60, Santa Cruz). Secondary antibodies were goat anti-donkey, goat anti-rabbit or isotype specific anti-mouse conjugated to Alexa Fluor 488 (Thermo Fisher Scientific). Cytkines were IL-2, IL-12 (Peprotech). Fluorescent markers were phalloidin Alexa Fluor 647, cell tracker violet (CTV), SNARF, LIVE/DEAD Fixable Near-IR (all from Thermo Fisher).

### Mice and Cell Lines

The *Sh2d2a*−/− C57BL/6 and Id-specific TCR transgenic BALB/c mice were previously described^19^. Briefly, *Sh2d2a*−/− mice on a mixed C57BL/6–129 background were backcrossed to C57BL/6 or BALB/c mice. *Sh2d2−/*+ BALB/c mice heterozygous for Id-specific TCR were crossed with *Sh2d2a−/*+ BALB/c mice to generate littermates both for studies of Id-specific TCR transgenic mice and normal BALB/c mice with or without *Sh2d2a*. The BALB/c MHC class II positive B cell lymphoma cell line A20 and F9 (A20 cells stably expressing Id^+^ λ2^315^ L chain^55^) were cultured in complete medium (RPMI 1640 medium supplemented with 10% fatal calf serum (FCS), 1 mM non-essential amino acids, 1 mM sodium pyruvate, 1 mM L-glutamine, 100 units/ml penicillin, 100 µg/ml streptomycin (all from GIBCOBRL®, Life Technologies™) and 50 µM β-mercaptoethanol (Sigma)).

### Purification of CD4+ T Cells

Human CD4+ T cells were isolated from PBMC using Dynabeads® CD4 Positive Isolation Kit, (ThermoFisher). Murine CD4+ T cells were isolated from single cell suspensions of spleens using Dynabeads® Untouched™ Mouse CD4 Cells Kit (ThermoFisher). The composition of the recovered population was more than 90% CD4+ T cells as analysed by flow cytometry.

### *In vitro* CD4+ T Cell Stimulation

Human CD4+ T cells were loaded with CTV before being stimulated with plate bound anti-CD3 (OKT3, 5 µg/ml) and soluble anti-CD28 (CD28.2, 1 µg/ml) in complete medium containing 30 U/ml IL-2 for 4 days. Cells were then stained with anti-TSAd-DyLight 488 and analysed by flow cytometry. Dividing cells were identified by CTV dilution. Murine CD4+ T cells were stimulated with Dynabeads® Mouse T-Activator CD3/CD28 beads (ThermoFisher), bead: cell ratio = 1:1 in complete medium containing 30 U/ml IL-2. CD3/CD28 beads were removed after 3 days *in vitro* and cultured in the presence of IL2 (30 U/ml) for another 7 days. Live cells were counted by trypan blue dye exclusion using a TC20 automated cell counter (Bio-Rad), and phenotyped by flow cytometry at 0, 3, 7 and 9 days *in vitro*. Surface staining was performed using CD4-PerCP-Cy5.5, CD44-FITC and CD62L-PE in FACS buffer (2% FCS, 0,1% sodium azide in PBS) at 4 °C. Cells were stained subsequently with LIVE/DEAD Fixable Near-IR to exclude dead cell, fixed for 10 minutes with 2% PFA in FACS buffer, and acquired on a FACSCantoII. Data was analysed using FlowJo version 7.6 (Tree Star Inc.), where live cells were gated on CD4-PerCP-Cy5.5 followed by analysis of CD44-FITC and CD62L-PE.

### Th1 differentiation assay

Id-specific TCR transgenic Th1 cells were obtained as previously described^27^. Briefly, splenic CD4+ T cells were cultured with 10 μg/mL anti–IL-4, 20 ng/mL IL-12 together with irradiated wild type splenocytes loaded with Id peptide as APC (1:3 T cell to APC ratio). The Id-peptide comprises residues 91–101 of the λ2^315^ L chain corresponding to CDR3. Mutated residues 94, 95, 96 are crucial for I-E^d^-restricted stimulation of Id-specific TCR transgenic CD4+ T cells^56,57^. Live cells were counted using a TC20 automated cell counter as above, at 0, 2, 5, 7 and 10 days *in vitro* before being phenotyped as described above on day 10.

### Conjugation assay

CD4+ T cells from Id-specific TCR transgenic BALB/c mice expanded for 5 days using CD3/CD28 beads, were rested for 48 hours in the absence of beads before being stimulated with irradiated (2500 rad) F9 or A20 cells. F9 cells presenting Id-peptide on MHC II strongly activates Id-specific TCR transgenic CD4+ T cells^22^. CD4+ T cells were labelled with 0,1 µM SNARF as per manufacturers instructions. The parental A20 cell line was used as a negative control. 1 × 10^6^ A20 or F9 target cells were co-cultured with 0,6 × 10^6^ Id-specific T cells in complete medium in 96 well U-bottom plates. Cells were centrifuged at 70 × g for 1 minute and incubated for indicated time points at 37 °C before stimulation. All subsequent pipetting was done gently with wide bore 200 µl pipette tips (VWR). Cells were stained with LIVE/DEAD Fixable Near-IR before being fixed with 2% PFA for 10 minutes, or fixed and permeabilised for 5 minutes with Acetone at −20 °C in case of γ–tubulin staining, followed by GB113-PE staining which binds Id-specific TCR (mAb; GB113^54^), at 10 µg/ml in FACS buffer for 30 minutes. Cells were then permeabilised and stained with FACS buffer containing 0,1% Saponin, 6,25U/ml Phalloidin Alexa Fluor 647 in combination with 1 µg/ml of one of the following antibodies: PAR3, PKCξ, PKCθ, Scrib, SAP97 (Santa Cruz), anti-γ–tubulin (Sigma) or IFNγ-FITC (BD). Cells were then washed and stained when necessary with secondary antibody goat anti-donkey, goat anti-rabbit or isotype specific anti-mouse conjugated to Alexa Fluor 488 (Thermo Fisher Scientific) together with DAPI. Cells were washed and stored in PBS, 0,1% NaAzide at 4 C until run on ImageStream X.

### Imagestream acquisition and analysis

Samples were acquired at 40x magnification on a four-laser, twelve channel, ASSIST calibrated ImageStream X (Amnis, Seattle, WA) imaging flow cytometer. 405 nm, 488 nm, 561 nm and 658 nm laser excitations were set to avoid pixel saturation. Single stained controls were collected and used to generate a compensation matrix to correct for spectral crosstalk. Data was analysed using IDEAS v6.1, where the T cell mask was defined based on the signal of the cell tracker SNARF. Live cells were identified by viability dye exclusion, and GB113 positive cells identified Id-specific TCR transgenic T cells. The aspect ratio and area of the DAPI signal was used to identify images containing two cells^58^. Selecting images with a high SNARF (T cell) intensity and aspect ratio was then done to identify images containing 1 T cell^58^. Using the “Interface” masking function in IDEAS^58^ the T cell synapse of the T cell was defined in all the images (Fig. 2A). Marker polarisation towards the synapse was quantified by calculating the average fluorescent signal in the synapse masks as a ratio to the average fluorescent signal of the whole T cell. To adjust for cell-to-cell variation in the size of the synapse mask, the signals were normalised against the area of the respective masks. A threshold ratio above 1,5 was defined as the fluorescent marker being polarised to the IS, based on the polarisation ratio of the uniformly distributed cell tracker SNARF (not shown).

### Statistical analysis

The data was exported and graphed using GraphPad Prism 6 (GraphPad Software, Inc.). To assess statistical significance, a two-tailed Student’s t-test was applied to compare two normally distributed datasets while two-way analysis of variance was applied to datasets with multiple time points. A significance level of 0,05 was used.
